# Investigating learning processes through analysis of navigation behavior using log files

**DOI:** 10.1007/s12528-023-09372-3

**Published:** 2023-04-27

**Authors:** Kerstin Huber, Maria Bannert

**Affiliations:** TUM School of Social Sciences and Technology, Department Educational Sciences, Chair for Teaching and Learning with Digital Media, Technical University of Munich, Arcisstr. 21, 80333 Munich, Germany

**Keywords:** Learning processes, Navigation behavior, Log files, Computer-based learning environments, Process mining

## Abstract

The empirical study investigates what log files and process mining can contribute to promoting successful learning. We want to show how monitoring and evaluation of learning processes can be implemented in the educational life by analyzing log files and navigation behavior. Thus, we questioned to what extent log file analyses and process mining can predict learning outcomes. This work aims to provide support for learners and instructors regarding efficient learning with computer-based learning environments (CBLEs). We evaluated log file and questionnaire data from students (*N* = 58) who used a CBLE for two weeks. Results show a significant learning increase after studying with the CBLE with a very high effect size (*p* < .001, *g* = 1.71). A cluster analysis revealed two groups with significantly different learning outcomes accompanied by different navigation patterns. The time spent on learning-relevant pages and the interactivity with a CBLE are meaningful indicators for Recall and Transfer performance. Our results show that navigation behaviors indicate both beneficial and detrimental learning processes. Moreover, we could demonstrate that navigation behaviors impact the learning outcome. We present an easy-to-use approach for learners as well as instructors to promote successful learning by tracking the duration spent in a CBLE and the interactivity.

## Theoretical background

The COVID-19 pandemic has demonstrated clearly that constant feedback and evaluation of the learning process, as well as progress in computer-based learning environments (CBLEs), pose challenges to teachers and learners alike (Grewenig et al., 2021). It has been extensively researched that monitoring and regulating the learning process and the progress of learners are essential to successful learning outcomes (Azevedo & Gašević, 2019; Hattie, 2017; McLaughlin & Yan, 2017; Schneider et al., 2021). Thus, the learning process needs to be tracked and evaluated before it can be customized to individual learners, especially whenever CBLEs are used (Arguel et al., 2017; Paans et al., 2020).

Real-time measures, such as log files (Reimann et al., 2014), physiological data (Malmberg et al., 2019), think-aloud protocols (Lim et al., 2021), and eye-tracking (Fan et al., 2022), have already proven beneficial as a way of analyzing learning processes; and they amount to a promising approach to providing instruction on a much-needed individual basis (Dindar et al., 2019; Goldman, 2009; Malmberg et al., 2019; Winne & Perry, 2000). However, in practice, these approaches have still not been implemented fully at educational institutions (Schneider et al., 2021).

The present work aims to show the extent to which log files can measure learning processes, as well as the impact that navigation behavior can have on learning. We present an easy-to-use method, which can be implemented in daily interactions with CBLEs.

### Literature Review

Collecting log files and exploring navigation behavior is a simple-to-implement, efficient method for tracing a learner’s activity and interactions in CBLEs (Arguel et al., 2017; Cerezo et al., 2020; Huang & Lajoie, 2021; Matcha et al., 2019).

Monitoring the learners’ interactions with a CBLE can provide insights into patterns of navigation behavior, which can influence feedback and teaching methods (Arguel et al., 2017; Azevedo & Gašević, 2019; Paans et al., 2020). This information can be gathered through log files and process mining, which are methods explored in the present work.

#### Measuring learning processes using log files

Log files record every interaction in a CBLE with a timestamp or the time spent on a particular page. Therefore, it is possible to analyze, for example, if the learner has carried out a specific task or read a learning-relevant text. In addition, the timestamp allows detection of how long the learners took for these activities. From this, it can be concluded that log files allow insights into individual learning and navigation behaviors (Azevedo et al., 2013; Malmberg et al., 2010). For example, systematic navigation behavior (frequent visits to learning-relevant pages) is positively correlated with increased knowledge (Bannert, 2006; Bannert et al., 2015). Lim and colleagues (2021) have shown that successful students re-read a learning-relevant text significantly more frequently than less successful students. Hence, navigation behavior is an indicator of learning outcomes.

Moreover, research has shown that monitoring of the learning process and Transfer performance correlate positively even after three weeks (Bannert et al., 2015; Sonnenberg & Bannert, 2015), which indicates that categories of learning (i.e., Recall, Comprehension, Transfer) correspond to navigation behavior. Furthermore, these categories are a crucial guideline for instructors when planning instructions and formulating learning goals (Anderson & Krathwohl, 2001; Krathwohl, 2002). For example, instructors can use flashcards to measure whether learners recall specific information. Writing a summary or blog journaling can be applied to measure if learners understand a concept. Additionally, instructors can instruct the learners to discuss an application example in chatrooms to determine if they can transfer their knowledge to new subjects (Churches, 2008). In order to explore if navigation behaviors reflect a specific category of the learning process, we developed a knowledge test, which measures each category but can also be summed up as a total learning score (see "Measures" section). Since these categories are structured from simple to complex (Bloom et al., 1956), we use the term *difficulty levels*, with the category *Recall* as the easiest, *Comprehension* as the intermediate, and *Transfer* as the hardest difficulty level.

Although log files are not as fine-grained as think-aloud data, they are an objective, automated measure. Thus, the learning process can be monitored without disturbing the learner (Hadwin et al., 2007; Winne, 2013). To analyze the sequence of events tracked in log files, we conducted a process mining model.

#### Describing the learning process using process mining

A popular analytical method for detecting patterns in navigation behavior is process mining (e.g., Lim et al., 2021; Sonnenberg & Bannert, 2016, 2019). Here, a process model is generated from log file data, which visualizes the interactions within the CBLE, based on specific events, revealing possible patterns of navigation behavior (Bannert et al., 2014). Based on these patterns, different groups of learners or learning strategies can be identified (e.g., Bannert et al., 2014; Huang & Lajoie, 2021; Matcha et al., 2019); thereby leading to the identification of either beneficial or detrimental learning behavior. This identification would make it possible to give individual, adequate feedback on the spot.

Consequently, we use navigation behavior to identify learning processes and also to ascertain whether it is possible to define groups of learners. We use log files to attain in-depth insights into learning behavior in combination with pre-post data (i.e., knowledge tests before and after learning). In view of the fact that we sought to present implementation in an everyday educational setting, we have evaluated data from a real seminar course.

## Methods

The general question with which this study is concerned is which in-depth insights log files provide regarding learning behavior in a real seminar course, in conjunction with pre-post questionnaire data. Hence, we devised the following research questions and hypotheses:

- To what extent can navigation behavior predict learning outcomes? (RQ1)
  - Navigation behavior affects learning outcomes. (H1)
  - Navigation behavior reflects the difficulty level of the learning process. (H2)
- To what extent do learners differ, based on navigation and learning behaviors? (RQ2)
  - Learners with high learning outcomes display different patterns of navigation than learners with low learning outcomes. (H3)

To answer the research questions formulated, we monitored and evaluated a unit (14 days long) of long-term use over ten weeks of a CBLE in a real seminar at the University of Saarland in Germany. This seminar lasted from May until July 2020 and consisted of four online meetings and four learning units. The online meetings took place every two or three weeks, followed by intermediate self-studying phases. Each learning unit included a self-studying phase and a subsequent online meeting. We evaluated one learning unit consisting of a 14-day self-studying phase and one online meeting. The tasks for the self-studying phases were to acquire the respective content, read an additional scientific article, and write a summary about it; which then had to be uploaded for monitoring purposes. The CBLE represented the learning material during self-study phases, while the online meetings served as an opportunity to pursue dialogue with the teacher and to clarify any potential questions. There were no instructions for the students on logging in and out of the CBLE. Moreover, the study time was not prescribed by the teacher. Hence, the students had the freedom and flexibility to learn according to their preferences (i.e., when and for how long they wanted to learn).

The teacher introduced the CBLE, the procedure, and the seminar topics in the first online session. Subsequently, students filled in a pre-test to measure their prior knowledge regarding the topics addressed; this included a declaration of consent and information on the processing and retention of personal data. After the meeting, the first phase of self-study started. These online meetings and the self-study sequences were repeated four times in relation to four topics. In each online meeting, students completed the test of knowledge concerning the previously learned topic. For further data analyses, we focused on one particular topic, which showed the most significant increase in learning and a sufficient sample size. Moreover, it proved possible to reduce the inherent complexity of the process mining model used.

### Participants

The participants consisted of 62 teacher-training students at the University of Saarland with a mean term time of 5.52 semesters (SD = 1.83) and with 41 females, 19 males, and one transgender individual. The mean age of the students was 22.18 years (SD = 2.51). Because four students did not complete the knowledge test after the self-study phase, only 58 participants could be included in the analysis of learning performances.

### Learning environment

As the CBLE, the teacher used the *Toolbox TeacherEducation* (TTE), a German openly available, multimedia, and interactive learning platform for teacher-training students (Lewalter et al., 2018a). This contains scientific summaries of miscellaneous topics in teacher training (e.g., psychological - feedback; didactical - problem-solving, or subject-specific - Pythagorean theorem), video tutorials, staged videos about different teaching units, and tasks or questionnaires. The TTE has been used in real seminar courses since 2018 and is evaluated constantly to ensure that its use and content contribute to successful learning (Lewalter et al., 2018b, 2020, 2022; Titze et al., 2021).

### Measures

The questionnaire (pre- and post-test) consists of 12 content-related multiple-choice items categorized in three difficulty levels based on Bloom’s Taxonomy (1956): Recall, Comprehension, and Transfer (see  "Literature Review"section), and the hierarchically ordered *Thinking Skills* (Anderson & Krathwohl, 2001; Churches, 2008). The *Recall* level stands for remembering or recognizing facts. The *Comprehension* level refers to understanding and paraphrasing an issue. The *Transfer* level relates to designing and planning a new structure (Churches, 2008). Each difficulty level was measured in terms of four items (examples see Table 1). The total score is 58 points (Recall 20 points, Comprehension 20 points, Transfer 18 points). Using this classification, we were able to measure and distinguish between different skills.

The students were instructed that one or, indeed, none of the items might potentially be correct. In order to reduce the guess probability, each item offers the optional response “*I don’t know*”. The test was designed and validated, based on previous evaluations. It features an average Cronbach’s alpha of 0.49, which is adequate, given that the questionnaires are designed explicitly for the TTE (see Schmitt 1996; Taber, 2018). Since the TTE deals extensively with certain areas, we did not expect a high level of reliability overall (see Berger & Hänze 2015).

**Table 1:** Example items for each difficulty level

Difficulty level	Item example
1: Recall	*“What are “open teaching” methods?”*
2: Comprehension	*“The teacher prepares a lesson in which learners have a high degree of choice regarding methods, media, and social format. Which approach does the teacher adopt?”*
3: Transfer	*“A teacher wants to use “tutorial learning” as a form of adaptive teaching. What should the teacher pay attention to?”*

The navigation behavior of the students was logged using the plugin *matomo* (https://matomo.org). Here, visits, visit durations, user ID, actions, page URLs, actions per visit, downloads, searches, transitions, and more can be tracked. We used duration, actions, page URLs, and user IDs for further data processing.

### Data analysis procedure

The log files generated from *matomo* included user ID, duration, type of activity (e.g., click or download), page URL, page title, and a timestamp. The remaining variables were not used for further data analyses. To obtain a clearer picture, we labeled every page of the TTE with a simple acronym, which indicated the topic and the page order (e.g., topic 1, page 4 = t1_p04). Moreover, we categorized the pages into *learning-relevant* (pages with learning-related content, depending on the topic), *orienting* (i.e., dashboard, profile, settings, home), *learning-irrelevant* (text, videos, or tasks about topics unrelated to learning) and *videos* (pages that show videos exclusively).

We developed a Python script for automated data analysis of the following steps. We aggregated the time spent on the categorized pages and this resulted in the variables *learning-relevant*, *learning-irrelevant, orienting*, and *videos* for each visit. Next, we summarized each log file per student. This resulted in a data set, which included all of the students and the respective duration, learning-relevant, orienting, learning-irrelevant times, and videos. We processed the log files for process mining techniques, using the pm4py Python package. The resulting output included a visit ID (user ID and visit count), activity (page acronym), and a timestamp (duration spent on the page). For process mining, we used the software application *Disco* from Fluxicon (https://fluxicon.com/disco/).

## Results

**Fig. 1:**
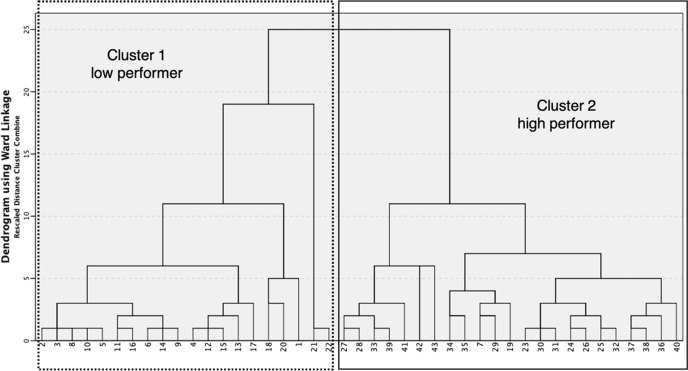
Dendrogram visualizing two meaningful cluster

**Fig. 2:**
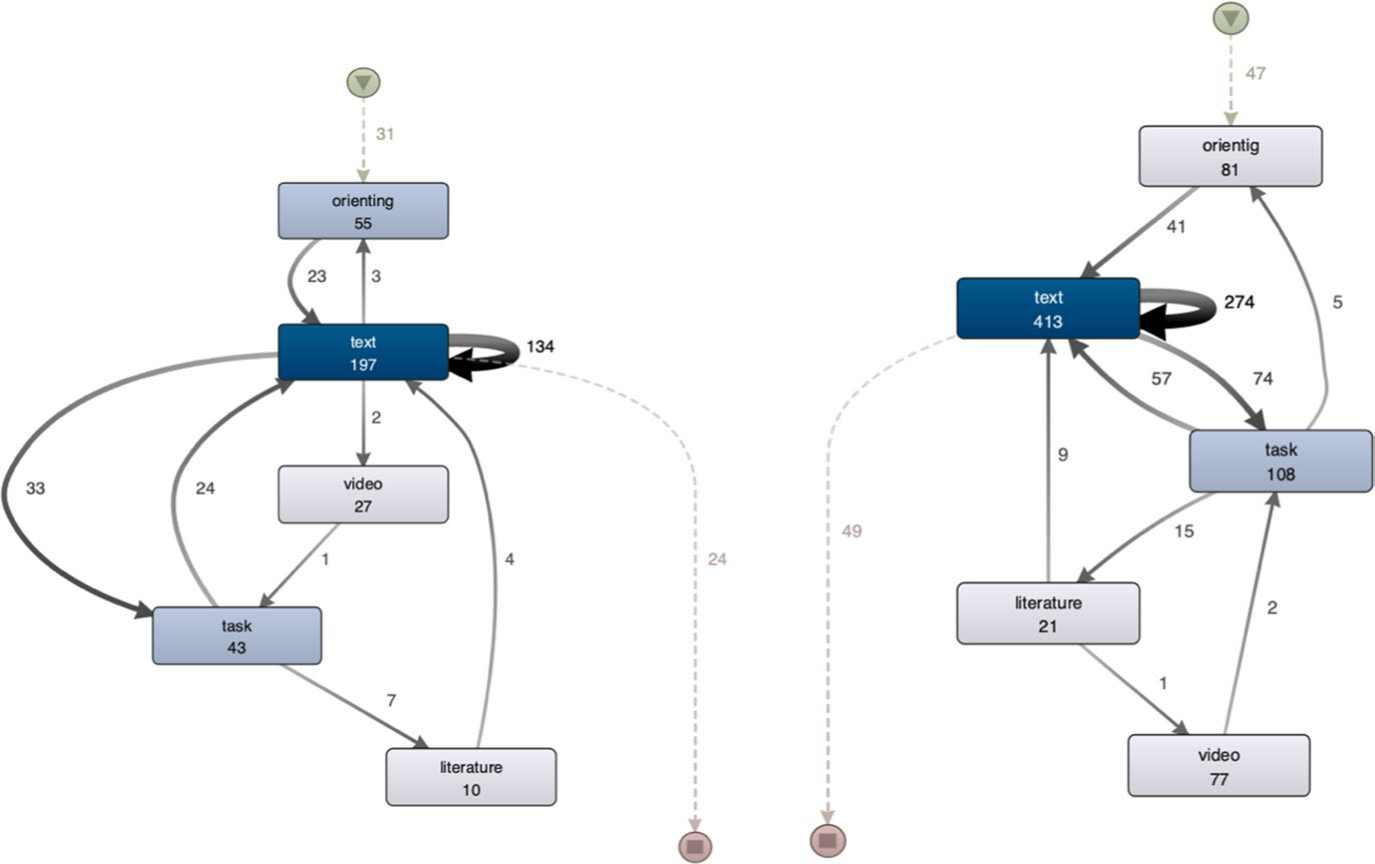
Process model for low performers (left) and high performers (right)

The following results are clustered, based on our data analysis procedure. Initially, we present descriptive data and a declaration of essential variables (see Tables 2 and 3). Additionally, we ran a paired samples t-Test (prior knowledge - learning outcome) to examine whether knowledge increased significantly after studying with the TTE. Afterwards, we present results from bivariate correlations to confirm H1, H2, and to answer RQ1 (see Table 4). Next, we show a hierarchical cluster analysis, including a One-Way ANOVA, in order to address H3 and RQ2 (see Table 5; Fig. 1). Based on the resulting clusters, we present our process mining approach, to give an exhaustive answer to RQ1 and RQ2 (see Tables 6 and 7, and Fig. 2).

**Table 2:** Important variable names and their declaration

	Variable name	Declaration
Prior knowledge	Pre-Difficulty Level 1	Total pre-test score for questions for Recall (see Table 1)
	Pre-Difficulty Level 2	Total pre-test score for questions for Comprehension (see Table 1)
	Pre-Difficulty Level 3	Total pre-test score for questions for Transfer (see Table 1)
	Pre-test score	Total pre-test score for all questions
Learning outcome	Difficulty Level 1	Total post-test score for questions for Recall (see Table 1)
	Difficulty Level 2	Total post-test score for questions for Comprehension (see Table 1)
	Difficulty Level 3	Total post-test score for questions for Transfer (see Table 1)
	Post-test score	Total post-test score for all questions
Navigation behavior (NB)	Duration (s)	Time spent in the learning environment
	Actions	Number of mouse clicks within the learning environment
	Learning-relevant (s)	Time spent on pages where theoretical basics are defined (e.g., models, state of research, concepts)
	Orienting (s)	Time spent on pages that serve the orientation in the learning environment (e.g., overview, profile)
	Learning-irrelevant (s)	Difference value of duration - time learning-relevant
	Videos (s)*	Time spent on pages where solely videos are displayed

*Note.* Time and duration variables are measured in seconds

*Only relevant for cluster analyses.

At first, we identified any outliers, using z-scores, resulting in different sample sizes (see Table 3). The mean score for overall prior knowledge (i.e., the pre-test score) was 32.98 and, therefore, above half of the maximum achievable score of 58 (see Table 3). Equally, the mean scores for Difficulty Levels 1 (*M* = 12.05) and 2 (*M* = 11.57) were above half the maximum score of 20. The mean score for Difficulty Level 3 was 8.74 and almost half the maximum achievable score of 18 (see Table 3). The most significant increase of 5.16 points was measured for Difficulty Level 3 and the smallest increase of 1.38 points for Difficulty Level 2. The knowledge gain in total was 8.67 points.

To analyze if the knowledge scores increase from prior knowledge (i.e., pre-test score) to the learning outcome (i.e., the post-test score) was significant, we carried out a paired samples t-Test. The results showed that the scores for all three Difficulty Levels (Level 1: *t*(41) = 6.26, *p* < .001, *g* = 1.12; Level 2: *t*(41) = 2.67, *p* = .005, *g* = 0.55; Level 3: *t*(41) = 10.43, *p* < .001, *g* = 2.05) and the scores in total (*t*(40) = 8.06, *p* < .001, *g* = 1.71) increased significantly with medium to very high effect sizes.

**Table 3:** Descriptive data for learning outcome and navigation behavior

	Variable	n*	Min	Max	*M*	*SD*
Prior knowledge	Pre-Difficulty Level 1	42	3	18	12.05	2.95
	Pre-Difficulty Level 2	42	4	16	11.57	3.05
	Pre-Difficulty Level 3	43	3	13	8.74	2.85
	Pre-test score	41	13	42	32.98	7.65
Learning Outcome	Difficulty Level 1	43	10	20	14.95	2.35
	Difficulty Level 2	43	7	19	12.95	2.65
	Difficulty Level 3	42	8	17	13.90	2.15
	Post-test score	43	28	56	41.65	5.61
Navigation Behavior (NB)^a^	Duration (s)	43	42	11,289	4298	3155
	Actions	43	0	53	22.74	13.23
	Orienting (s)	44	32	3263	1009	891.9
	Learning-relevant (s)	44	0	8821	2999	2460
	Learning-irrelevant (s)	43	32	3453	1213	985.5

*Note.* Time and duration variables are measured in seconds

*Outliers were identified and excluded for further data analyses, resulting in different sample sizes

The maximum score for learning outcome is 58 in total

The maximum score for Difficulty Levels 1 and 2 is 20 and for Difficulty Level 3, 18

^a^The variable “video” is not included here because it was only relevant for cluster analyses

Because our first hypothesis (see "Methods" section) addresses the influence of the navigation behavior (i.e., duration, actions, orienting, learning-relevant, learning-irrelevant) and learning outcome (i.e., post-test score), we analyzed the relationships between these constructs (see Table 4). The variable “video” is not included here because it was only relevant for cluster analyses. The bivariate correlation analysis indicated that, except for the variables orienting and learning-irrelevant, navigation behavior has a positive linear relationship with the post-test score. Thus, navigation behavior affects the learning outcome (see Table 4; results can be seen in the second row). However, prior knowledge has no significant relationship with navigation behavior (see Table 4; results can be seen in the first row).

To address the more detailed H2, we examined the correlations described for each Difficulty Level separately. In this way, it was possible to map out the difficulty level of the learning process (see "Literature Review" section). Results show that Difficulty Level 2 does not correlate with any variable relating to navigation behavior. However, the variables duration, actions, and learning-relevant have a positive linear relationship with Difficulty Levels 1 and 3. The variables orienting and learning-irrelevant do not correlate with learning outcome (see Table 4).

**Table 4:** Results for correlation analysis of learning outcome and navigation behavior

	Duration^a^		Actions^a^		Orienting^b^		Learning-relevant^b^		Learning-irrelevant^b^	
	*p*	*r*	*p*	*r*	*p*	*r*	*p*	*r*	*p*	*r*
Pre-test Score	0.378	− 0.143	0.116	− 0.252	0.789	− 0.043	0.279	− 0.173	0.875	0.026
Post-test Score	0.028	0.338^*^	0.028	0.339^*^	0.053	0.297	0.007	0.404^**^	0.472	0.114
Difficulty Level 1	0.021	0.356^*^	0.009	0.398^**^	0.232	0.186	0.006	0.410^**^	0.229	0.190
Difficulty Level 2	0.238	0.186	0.073	0.280	0.064	0.285	0.069	0.280	0.924	0.015
Difficulty Level 3	0.017	0.371^*^	0.049	0.309^*^	0.096	0.260	0.017	0.366^*^	0.567	0.092

*Note.* Pearson correlation, 2-tailed, ** *p* < .01, * *p* < .05

^a^*n* = 42

^b^*n*= 43

Since log files contain a large quantity of data and we consider our dataset promising, we wanted to zoom in and explore it in greater detail. The analyses performed above, which are rather conservative, were unable to uncover the dynamic, individual character of navigation behaviors. Therefore, we conducted a hierarchical cluster analysis, using the Ward Linkage; which generated highly homogeneous clusters (see also Huang & Lajoie 2021; Paans et al., 2020; see Fig. 1).

In order to detect distinct groups of learners (see H3), we included all outliers in the data set. Only one participant had to be excluded due to missing post-test data, resulting in a sample of *N* = 43. The dendrogram from the hierarchical cluster analysis showed two meaningful clusters (see Fig. 1; cluster 1: *n* = 20, cluster 2: *n* = 23). The x-axis represents the anonymized number of each participant. The y-axis shows the distance between each cluster procedure (see Fig. 1). For the analysis, z-scores were used, though the dendrogram presents raw scores. We chose the Euclidean distance as the distance measure; and, due to the sample size, we predefined two clusters, in order to get a clear division. We used the navigation behavior (i.e., duration, actions, orienting, learning-relevant, learning-irrelevant, and videos) as cluster variables. Based on this procedure, we managed to identify two distinct groups (see Fig. 1). Since this division seemed sufficient and was of a similar sample size, we proceeded with the suggested clusters. Afterward, we conducted a variance analysis to examine how the groups differ based on their navigation behavior and learning outcomes and whether this difference is significant (see Table 5).

Both groups differ significantly in their navigation and learning behaviors (see Table 5). Noteworthy is the fact that the first cluster had consistently fewer values for all variables (Fig. 1, left side, and Table 5). However, because the prior knowledge in this case was not significantly different from the “better” group, we named the first cluster *low performers* and the group with higher values *high performers*.

The low performers showed a significantly lower learning outcome on all three Difficulty Levels, especially on Level 1 and 3, with very high effect sizes (see Table 5). The group differences could also be measured in the navigation behavior. The high performers spent more than twice as much time in the TTE on learning-relevant, orienting, and video pages and performed almost twice as many actions as the low performers. However, the high performers also visited learning-irrelevant pages significantly longer than the low performers. Since the high performers showed a higher duration overall without significant differences for learning-irrelevant pages, this is not further questionable (see Table 5).

**Table 5:** Means, standard deviations, and one-way analysis of variance in low and high performers

Variable	Low performers^a^		High performers^b^		*F*(1, 41)	η^2^
	*M*	*SD*	*M*	*SD*		
Pre-test score	31.1	9.16	32.5	6.20	0.341	0.008
Difficulty Level 1	13.5	1.761	16.4	1.90	26.7***	0.394
Difficulty Level 2	11.7	2.03	14.0	2.69	10.2*	0.199
Difficulty Level 3	12.4	2.26	14.8	1.85	15.0***	0.268
Post-test score	37.6	4.50	45.2	3.74	37.2***	0.476
Duration	1914	2197	5583	2835	22.0***	0.349
Actions	15.7	12.6	31.3	12.1	17.2***	0.295
Orienting	516	598	1175	898	7.75*	0.159
Learning-relevant	1530	1745	4387	2220	21.5***	0.344
Learning-irrelevant	384	667	1196	1697	4.03	0.089
Videos	137	429	974	1499	5.80*	0.124

*Note.* ^*^p < .05, ^***^p < .001

^a^*n* = 20

^b^*n*= 23

Besides detecting different learner groups through log file and cluster analyses, we were interested in examining the sequence and flows of the navigation behavior of each learner group. Therefore, we conducted process mining analyses, which are a fruitful approach to reveal such sequential flows. Here, we used the log file data to create a process mining model based on the significantly different clusters that resulted. We summarized homogeneous pages (e.g., pages with text or tasks) to generate a transparent process model (see Table 6).

**Table 6:** Variables for process modeling and declaration

Variable name	Declaration
Text	Pages with theoretical basics, concepts, and models
Video	Pages with videos exclusively
Task	Pages with tasks/questionnaires
Literature	List of references
Orienting	Pages that serve the orientation in the learning environment

The results from the process analyses support the cluster analysis carried out and the emerging groups of low and high performers demonstrated (see Fig. 1; Table 5). The high performers visit text, video, task, and literature more than twice as often as the low performers. Moreover, high performers visit orienting pages more often than the low performers, though the difference is not as significant as with the other categories (see Tables 5 and 7; Fig. 2).

**Table 7:** Absolute and mean activity frequency for each category

Categories	Activity frequency				
	Low performers			High performers	
	Absolute value		*M*	Absolute value	*M*
Text	197		9.85	413	17.96
Video	27		1.35	77	3.35
Task	43		2.15	108	4.70
Literature	10		0.50	21	0.913
Orienting	55		2.75	81	3.52

After presenting the descriptive data, we exported the process model to illustrate low and high performers’ tread routes (see Fig. 2). Both group models start with orienting pages and walk along to text pages. It is noticeable that there is a loop on the process model showing the high performers. They go directly back and forth from the text to task pages, probably in order to verify their knowledge; and then they return to learning-relevant content on the text pages. Low performers, on the other hand, go directly to video, task, and literature pages without any major loop pattern (see Fig. 2).

## Discussion and implications

In this study, we investigated the extent to which log files and navigation behavior can predict learning outcomes. Moreover, we sought to show that learners with high learning outcomes display different navigation behavior than learners with low learning outcomes (e.g., Bannert 2006; Lim et al., 2021).

Our approach contributes to meeting the challenge of how instructors can monitor and evaluate the learning process and progress in CBLEs; and how they can give adequate feedback at the appropriate time, based on the learner’s needs (Paans et al., 2020; Schneider et al., 2021). Thus, our approach and findings can also support instructors and designers of CBLEs. Instructors can observe how learners interact with the CBLE and what individual learning style they prefer for effective learning. Thus, instructors can mitigate the absence of face-to-face interaction, given that immediate feedback is more effective (Bloom, 1984). Based on instructors’ observation of learners, beneficial learning methods can be introduced or promoted (Hillmayr et al., 2020; Van der Kleij et al., 2015). If a specific navigation pattern leads to low learning outcomes, the designer of the CBLE would be able to adapt the learning content, environment, or instructions in such a way as to ensure successful learning (Bousbia et al., 2010).

We hypothesized that navigation behavior affects the learning outcome. Therefore, we correlated the post-test score with navigation behavior. The results obtained show that the post-test score has a significant linear relationship with the variables - duration, actions, and learning-relevant (see Table 4). Thus, higher duration, especially on learning-relevant pages, as well as a greater number of actions, contribute to successful learning; which supports our first hypothesis. These results give rise to the conclusion that learners need to invest time on learning-relevant pages and engage actively with the CBLE to reach high-level learning outcomes; which is in line with the findings of Mayer (2014).

The pre-test score does not correlate significantly with the navigation behavior. Hence, prior knowledge does not affect navigation behavior.

Our second hypothesis addressed the relation between the difficulty levels of learning (Recall, Comprehension, Transfer) and navigation behavior. Here, we wanted to analyze if navigation behavior can reflect Recall, Comprehension, or Transfer performance (e.g., high Transfer performance goes along with a different navigation pattern than high Recall performance). In fact, we were able to show a significant linear relationship between Difficulty Levels 1 (Recall) and 3 (Transfer) and navigation behavior (i.e., duration, actions, learning-relevant, see Tables 1 and 4); which means that the longer the students stayed in the TTE and, especially, on learning-relevant pages, the better Recall and Transfer performance were. The time spent on orienting pages shows, as expected, no significant relationship with learning but, at the same time, does not negatively affect learning (see Table 4). Based on these results, our second hypothesis can also be supported.

Regarding our first research question, we were able to show that the time spent on learning-relevant pages and the associated interactivity (i.e., number of actions) are important factors for high learning outcomes. Moreover, here, conclusions regarding the level of difficulty can be drawn: The more extended learners stay on learning-relevant pages and the more intense their interaction (measured by actions) with the TTE, the better the Recall and Transfer performance. The implications for instructors are that the durations and actions within a CBLE are meaningful for successful learning. By marking learning-relevant pages in a CBLE and tracking the intensity of interactions, as well as the time learners spent in the CBLE, instructors can ensure high learning outcomes. Since these factors can be measured and evaluated quite rapidly, implementation in everyday education is, indeed, feasible.

We hypothesized that learners with high learning outcomes show a different navigation pattern than learners with a low learning outcome. To validate this third hypothesis and based on Huang and Lajoie (2021) and Paans and colleagues (2020), we implemented both a cluster analysis and a process model (see Figs. 1 and 2). The two resulting groups (low performers and high performers) differ significantly regarding the learning outcome and navigation behavior, which supports our third hypothesis. However, both groups show similar prior knowledge. A significant difference in Recall, Comprehension, and Transfer performance in favor of the high performers can be measured (see Table 5). Additionally, the high performers showed significantly higher durations on learning-relevant, orienting, and video pages (more than twice as long). Moreover, the high performers interacted more actively with the TTE (more than twice as many actions). Interestingly, the high performers also stayed more than twice as long on learning-irrelevant pages. However, this result is not unusual, since the high performers have an overall higher duration.

An explanation for the poor interaction of the low performers could be that, due to their high level of prior knowledge, they did not see the need to acquire the learning content.

Regarding our second research question, namely, the extent to which learners differ, as measured by navigation behavior and learning outcome, we included a process model designed to make possible navigation patterns visible. This model reveals that the high performers show higher activity frequencies for text, video, orienting, literature pages, and tasks implemented in the TTE (see Tables 6 and 7). Lim and colleagues (2021), as well as Bannert and colleagues (2014), showed that high-frequency activity leads to superior learning outcomes. More precisely, they identified specific self-regulated learning phases by categorizing the activities involved (e.g., orientation, planning, monitoring, search, evaluation; Bannert et al., 2014; Lim et al., 2021). Doing so makes the actions and interactions with the CBLE more specific regarding self-regulated learning.

The process model reveals that the high performers also have a different pattern of navigation behavior, which leads to a superior learning outcome. The high performers present a conspicuous looping pattern, including the text pages and the tasks, which gives rise to the conclusion that they read a text, test their knowledge by carrying out a task, and then return to studying.

As with the high performers, MacGregor (1999) found patterns in learners’ navigation behavior by conducting a cluster analysis: The “sequential studiers” are distinctive in terms of methodical strategy and focus on reading. Lawless and Kulikowich (1996) found users by performing a cluster analysis, which spent little time in the CBLE and did not use many features or inspected pages (“apathetic hypertext users”). This pattern is similar to our finding regarding the low performers.

In conclusion, we contributed to the research field of log file analyses and process mining approaches by showing that log files are a robust tool suited to obtaining information about the learning process in a CBLE. Additionally, we demonstrated that analyzing navigation behavior is a promising approach when it comes to predicting learning outcomes. We were able to demonstrate navigation behavior patterns that indicate both, beneficial (high performers) and detrimental (low performers) learning. Our work counters the absence of monitoring learners’ activity in a CBLE; and it does this by presenting a method that is easy to use, easy to evaluate and easy to integrate into daily educational routines. Thus, instructors can detect either beneficial or detrimental learning processes, as appropriate, and then provide adequate feedback.

## Limitations

Regarding learning-related variables, it is striking that the time learners spend on learning-irrelevant pages does not correlate negatively with learning outcomes. The actual generation of this variable provides an explanation: Given that we evaluated just one section of the entire semester, we needed to infer what was “learning-irrelevant” from within this section. Because as soon as we define every page beside the topic we analyzed as learning-irrelevant, a fuzzy and disproportionally large number remains. Thus, the variable learning-irrelevant could be inconclusive.

Our results show that Difficulty Levels 1 (Recall) and 3 (Transfer) are meaningful variables. Yet, Difficulty Level 2 (Comprehension) does not correlate with navigation behavior and thus, seems of no significance. The reason for this could be the nature of our self-designed questionnaire, which probably did not define the Comprehension category clearly enough. However, despite this, Recall and Transfer performance, as well as the overall post-test score, are meaningful indicators of knowledge gain and are sufficient for our purposes.

We mentioned the connection between self-regulated learning phases and activities within a CBLE in the discussion (see “Discussion and implications” Sect). Including self-regulated learning, various measures would present our variable actions more precisely and would yield information about learners’ cognitive processes. However, self-regulated learning and its impact on navigation behavior and learning outcomes have already been researched sufficiently (e.g., Bannert et al., 2014, 2015; Fan et al., 2022; Lim et al., 2021; Matcha et al., 2019; Schoor & Bannert, 2012; Sonnenberg & Bannert, 2015).

## Conclusion

Learning with CBLEs is indispensable and provides a host of benefits for learners. Learners can interact actively with learning content and study at their own pace and in their preferred learning style. Due to their static, unified setting, both traditional lectures and frontal teaching methods have recently been called into question. Studying with CBLEs counters these issues, because learners can acquire knowledge in line with their own needs (Goedhart et al., 2019; Estrada et al., 2019). However, instructors must track the learning process and learners’ progress itself, in order to ensure that specific learning goals are met.

Besides questionnaires, navigation behavior is a highly useful measure when it comes to tracking the learning process and associated progress (e.g., Bousbia et al., 2010; Matcha et al., 2019; Paans et al., 2019).

Our results show that both, log files and navigation behavior can predict learning outcomes: The time spent in a CBLE and the intensity of interactions with the CBLE yield information about Recall and Transfer performance. Furthermore, learners with a high learning outcome navigate differently through the CBLE (see Fig. 2): The high performers interact with the CBLE in a more frequent and intense manner. Moreover, the pattern of navigation behavior varies, depending on the learning outcome: High performers show a specific linkage between text and tasks (see Fig. 2).

Based on our research, log files and navigation behavior are validated to predict learning outcomes and the difficulty level of learning outcomes (Recall and Transfer performance). We were able to show that navigation behavior significantly impacts learning outcomes and that learners with high learning outcomes display significantly different navigation behavior than learners with low learning outcomes (see also Bannert 2006; Lim et al., 2021).

Our approach and results are promising, since we evaluated data from a real seminar course and successfully tested the feasibility of implementing the monitoring of learning processes in a CBLE.
